# Active secretion of a thermostable transglutaminase variant in *Escherichia coli*

**DOI:** 10.1186/s12934-022-01801-9

**Published:** 2022-04-29

**Authors:** Xinglong Wang, Beichen Zhao, Jianhui Du, Yameng Xu, Xuewen Zhu, Jingwen Zhou, Shengqi Rao, Guocheng Du, Jian Chen, Song Liu

**Affiliations:** 1 National Engineering Laboratory for Cereal Fermentation Technology, Jiangnan University, 1800 Lihu Road, Wuxi, 214122 Jiangsu China; 2 Science Center for Future Foods, Jiangnan University, 1800 Lihu Road, Wuxi, 214122 Jiangsu China; 3 The Key Laboratory of Carbohydrate Chemistry and Biotechnology, Ministry of Education, Jiangnan University, 1800 Lihu Road, Wuxi, 214122 Jiangsu China; 4 Jiangsu Provisional Research Center for Bioactive Product Processing Technology, Jiangnan University, 1800 Lihu Road, Wuxi, 214122 Jiangsu China; 5 College of Food Science and Engineering, Yangzhou University, Yangzhou, 214122 Jiangsu China

**Keywords:** *Streptomyces mobaraenesis*, Transglutaminase, Thermostability, Active expression, Protein secretion, *Escherichia coli*

## Abstract

**Background:**

*Streptomyces mobaraenesis* transglutaminase (smTG) is widely used to generate protein crosslinking or attachment of small molecules. However, the low thermostability is a main obstacle for smTG application. In addition, it is still hard to achieve the secretory expression of active smTG in *E. coli*, which benefits the enzyme evolution. In this study, a combined strategy was conducted to improve the thermostability and secretory expression of active smTG in *E. coli*.

**Results:**

First, the thermostable *S. mobaraenesis* transglutaminase variant S2P-S23V-Y24N-S199A-K294L (TGm1) was intracellularly expressed in pro-enzyme form in *E. coli*. Fusing the pro-region of *Streptomyces hygroscopicus* transglutaminase (proH) and TrxA achieved a 9.78 U/mL of intracellular smTG activity, 1.37-fold higher than the TGm1 fused with its native pro-region. After in vitro activation by dispase, the TGm1 with proH yielded FRAPD-TGm1, exhibiting 0.95 ℃ and 94.25% increases in melting temperature and half-life at 60 ℃ compared to FRAP-TGm1 derived from the expression using its native pro-region, respectively. Second, the TGm1 with proH was co-expressed with transglutaminase activating protease and chaperones (DnaK, DnaJ, and GrpE) in *E. coli*, achieving 9.51 U/mL of intracellular FRAPD-TGm1 without in vitro activation. Third, the pelB signal peptide was used to mediate the secretory expression of active TGm in *E. coli*, yielding 0.54 U/mL of the extracellular FRAPD-TGm1. A script was developed to shuffle the codon of pelB and calculate the corresponding mRNA folding energy. A 1.8-fold increase in the extracellular expression of FRAPD-TGm1 was achieved by the Top-9 pelB sequence derived from the coding sequences with the lowest mRNA folding energy. Last, deleting the gene of Braun’s lipoprotein further increased the extracellular yield of FRAPD-TGm1 by 31.2%, reached 1.99 U/mL.

**Conclusions:**

The stabilized FRAPD-smTG here could benefit the enzyme application in food and non-food sectors, while the *E. coli* system that enables secretory expression of active smTG will facilitate the directed evolution for further improved catalytic properties. The combined strategy (N-terminal modification, co-expression with chaperones, mRNA folding energy optimization of signal peptide, and lipoprotein deletion) may also improve the secretory expression of other functional proteins in *E. coli*.

**Supplementary Information:**

The online version contains supplementary material available at 10.1186/s12934-022-01801-9.

## Introduction

Transglutaminase (EC 2.3.2.13) can generate protein crosslinking or attachment of small molecules based on the acyl transfer between γ-carboxyamide group (acyl donor) and a primary amine (acyl acceptor) [1]. Compared with mammal and plant derived transglutaminases, the transglutaminase from *Streptomyces mobaraenesis* (smTG) was calcium-independent during the catalytic reaction and can be massively produced using microorganisms [2]. Thus, smTG was gradually adopted to replace mammal’s derived transglutaminase in the food industry to improve the texture properties of protein-based foods [3]. In addition, smTG also exhibits great potentials for antibody drug conjugation, tissue engineering, textile and leather processing [4–6]. The expanded utility horizon has brought out challenges for evolving smTG in order to meet the special needs, such as high temperature tolerance, catalytic activity, and selectivity. As a commonly used platform for enzyme evolution, *E. coli* showed many advantages, including it’s easy to be genetic modification and the rapid growth rate. Therefore, achieving the active expression of smTG in *E. coli* could benefit its production as well as the directed evolution for improved catalytic properties.

In *S. mobaraenesis*, transglutaminase is synthesized as a pro-enzyme form, which is activated by removing its N-terminal pro-region via transglutaminase activating protease (TAMEP), yielding the active transglutaminase with a FRAP tag (FRAP-smTG) [7]. Then, the FRAP tag was cleaved by an endogenous aminopeptidase [7]. It has demonstrated that the additional FRAP tag shows minor impact on both specific activity and thermostability of smTG [8]. Because the pro-region is important for smTG folding [9, 10], smTG was generally expressed in inactive form with its pro-region in *E. coli* and activated in vitro using a single protease, yielding the active FRAP-smTG [8, 11]. Obviously, the in vitro activation is unfavorable for fast activity detection during the directed evolution. Co-expression with site-specific proteases is a common strategy for achieving the active expression of smTG in *E. coli*. Researchers have used a 3C-protease digestion site to link pro-region and mature region of smTG, resulting in the in vivo activation by co-expressing the 3C protease [12]. In a recent study, Tobacco etch virus protease and its cleavage site were also used for actively expressing smTG [13]. However, the expression level of active smTG (0.2 U/mL/OD_600_) was still very low, and no active smTG could be secreted into the culture medium [12, 13], which makes the enzyme extraction harder. Therefore, it is desirable to improve the secretory expression of active smTG in *E. coli*.

It has been reported that the pro-region could regulate the protein yield of transglutaminase in addition to the protein folding. Fusing the pro-region of *Streptomyces caniferus* at N-terminus increased the expression of *Bacillus subtilis* transglutaminase in *E. coli* by 70% [14]. The expression enhancement of the smTG in *Corynebacterium glutamicum* was also observed after fusing with a chimeric pro-region from *Streptomyces cinnamoneus* transglutaminase [15]. In *E. coli*, co-expressing with chaperones can benefit enzyme folding process and soluble form [16, 17]. As mRNA contain stable elements that can regulate the translation rate and protein folding, and mRNA folding energy may thus be a key factor for protein expression [18]. Many reports have suggested that the secretory expression of recombinant proteins in *E*. *coli* was greatly affected by the outer membrane permeability [19]. To date, a series of those genes responsible for outer membrane formation has been identified in *E*. *coli*, such as Braun’s lipoprotein (*lpp*), olfactory marker protein (*omp*), and peptidoglycan-associated protein (*excC*) [20–22]. Deleting or over-expressing these genes has led to the improved extracellular expression of recombinant proteins in *E. coli* [20, 21]. In summary, pro-region substitution, over-expressing chaperones, mRNA regulation, and genetic modification of membrane protein genes could thus be the candidate tools to improve the secretory expression of active smTG in *E. coli*.

In this study, we firstly examined the effect of the pro-regions from different *Streptomyces* transglutaminases on the expression of a thermostable smTG variant S2P-S23V-Y24N-S199A-K294L (TGm1) in *E. coli*. Then, TAMEP and chaperones were co-expressed to achieve the in vivo activation of the pro-enzyme and the production enhancement, respectively. Signal peptide pelB was used for secretory expression, and the coding region of pelB was optimized according to the mRNA folding energy. The pelB variant contributed to the highest extracellular active TGm1 expression was selected, and used to guide TGm1 secretory expression in a *lpp* knockout *E. coli* strain. Since the activation changed N-terminal residual peptide of TGm1, we characterized the catalytic property and investigated the mechanism behind.

## Results and discussion

### Improving the expression of the pro-enzyme of TGm1 through N-terminal fusion

**Fig. 1:**
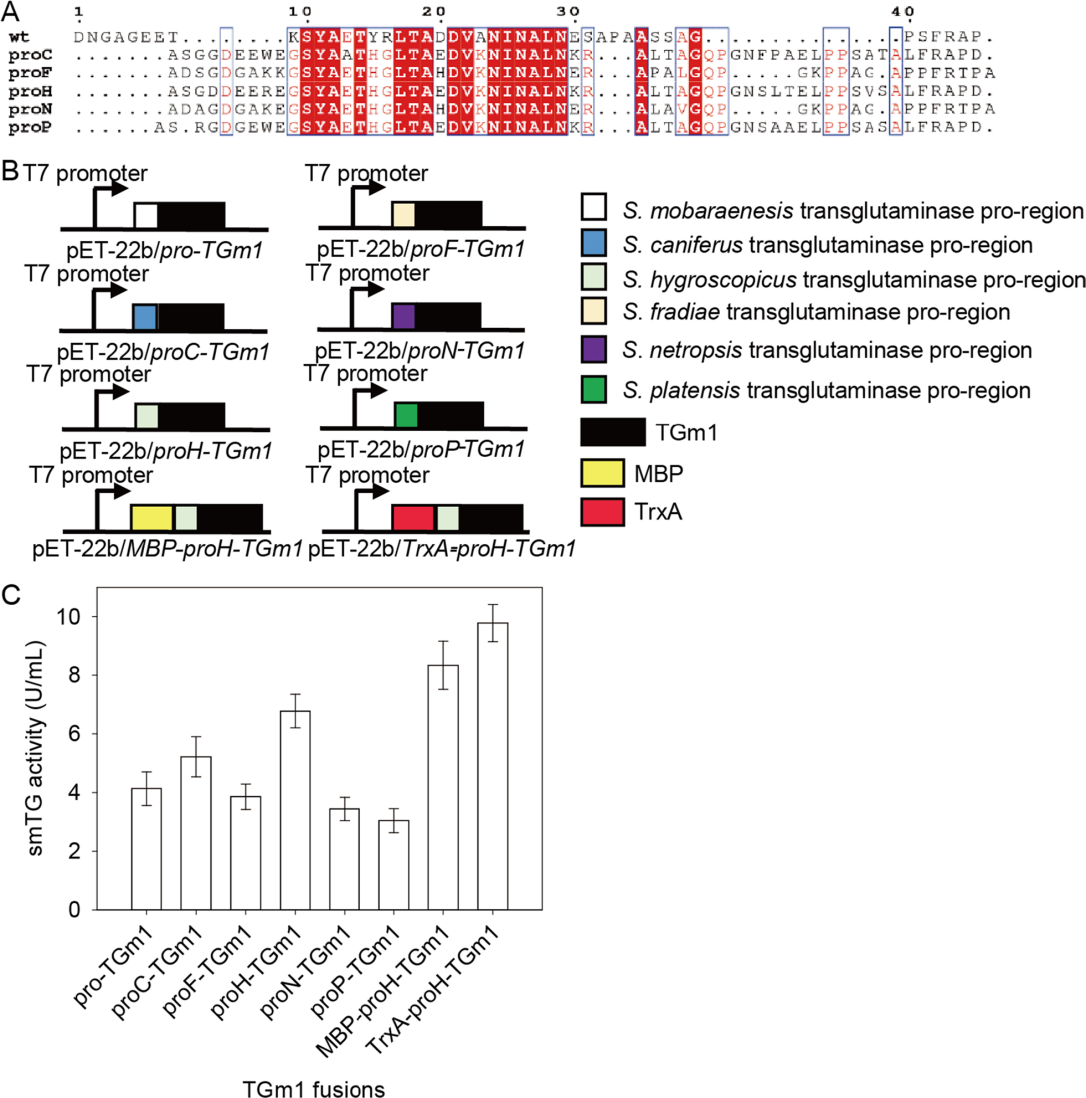
Expression of the recombinant TGm1 fused with different pro-region and expression tags in *E. coli*. **A** Amino acid sequence alignment of the pro-regions from different *Streptomyces* transglutaminases. pro: the pro-region from smTG; proC: the pro-region from *S. caniferus*; proF: the pro-region from *S. fradiae*; proH: the pro-region from *S. hygroscopicus*; proN: the pro-region from *S. netropsis*; proP: the pro-region from *S. platensis*. **B** The expression cassette of the TGm1 fused with different pro-regions and expression tags. **C** The intracellular smTG activity of the *E*. *coli* expressing the TGm1 with different pro-regions. Each recombinant *E. coli* was cultivated in TB medium at 20 ℃ for 32 h after 0.1 mM IPTG addition. The intracellular smTG activities of the recombinant strains were measured after in vitro activation by dispase at a final concentration of 2 mg/mL

To test the effects of the pro-region on the TGm1 expression in *E. coli*, we constructed a series of pET-22b derivatives expressing TGm1 fused with the transglutaminase pro-regions from *S. caniferus* (proC), *S. fradiae* (proF), *S. hygroscopicus* (proH), *S. netropsis* (proN), and *S. platensis* (proP), respectively (Fig. 1A, B). Then, each plasmid was transformed into in *E. coli* BL21 (DE3) and expressed in TB medium at 20 ℃ for 32 h by adding 0.1 mM IPTG. The intracellular smTG activities of the recombinant strains were measured after in vitro activation by dispase. As shown in Fig. 1C, the strains expressing proC-TGm1 and proH-TGm1 produced 5.22 and 6.78 U/mL of intracellular TGm1, 26% and 64% higher than that expressing the enzyme with its native pro-region, respectively. In agreement with previous studies [10, 14], our result showed that the pro-region substitution is an efficient strategy for regulating transglutaminase expression level in *E. coli*. To test the effect of soluble expression tags, we constructed the plasmids pET-22b/*MBP-proH-TGm1* (encoding the proH-TGm1 fused with MBP [23]) and pET-22b/*TrxA-proH-TGm1* (encoding the proH-TGm1 fused with TrxA [23]), respectively (Fig. 1B). After transformed in the *E. coli*, the intracellular smTG activity of the strain expressing pET-22b/*TrxA-proH-TGm1* reached 9.78 U/mL, which was 44% higher than that of the strain without fusing the expression tag (Fig. 1C). SDS-PAGE analysis indicated that the band thickness of TrxA-proH-TGm1 was also increased compared to that of proH-TGm1 (Additional file 1: Fig. S1). These results indicate that TrxA fusion could improve the expression of TGm1 in *E. coli*. However, fusing MBP slightly impacted the intracellular expression (Fig. 1C).

Sequence alignment revealed that the pro-regions examined were not highly conserved, especially in their C-terminal regions (Fig. 1A). Previous studies indicate that the smTG activated by dispase or TAMEP has an additional FRAP tag on its N-terminus [8, 24]. Because the C-terminus of the native pro-region of smTG and proH are respectively ended with FRAP and FRAPD, the pro-TGm1 and proH-TGm1 may also vary in the N-terminal region after in vitro activation. To conduct N-terminal sequence analysis, the two pro-enzymes were activated by dispase and purified by affinity chromatography (Additional file 1: Fig. S2). As expected, the additional N-terminal peptides of the activated pro-TGm1 and proH-TGm1 were FRAP and FRAPD, respectively (Additional file 1: Fig. S3).

### Improving the active expression of TGm1 through the co-expression with TAMEP and chaperones

**Fig. 2:**
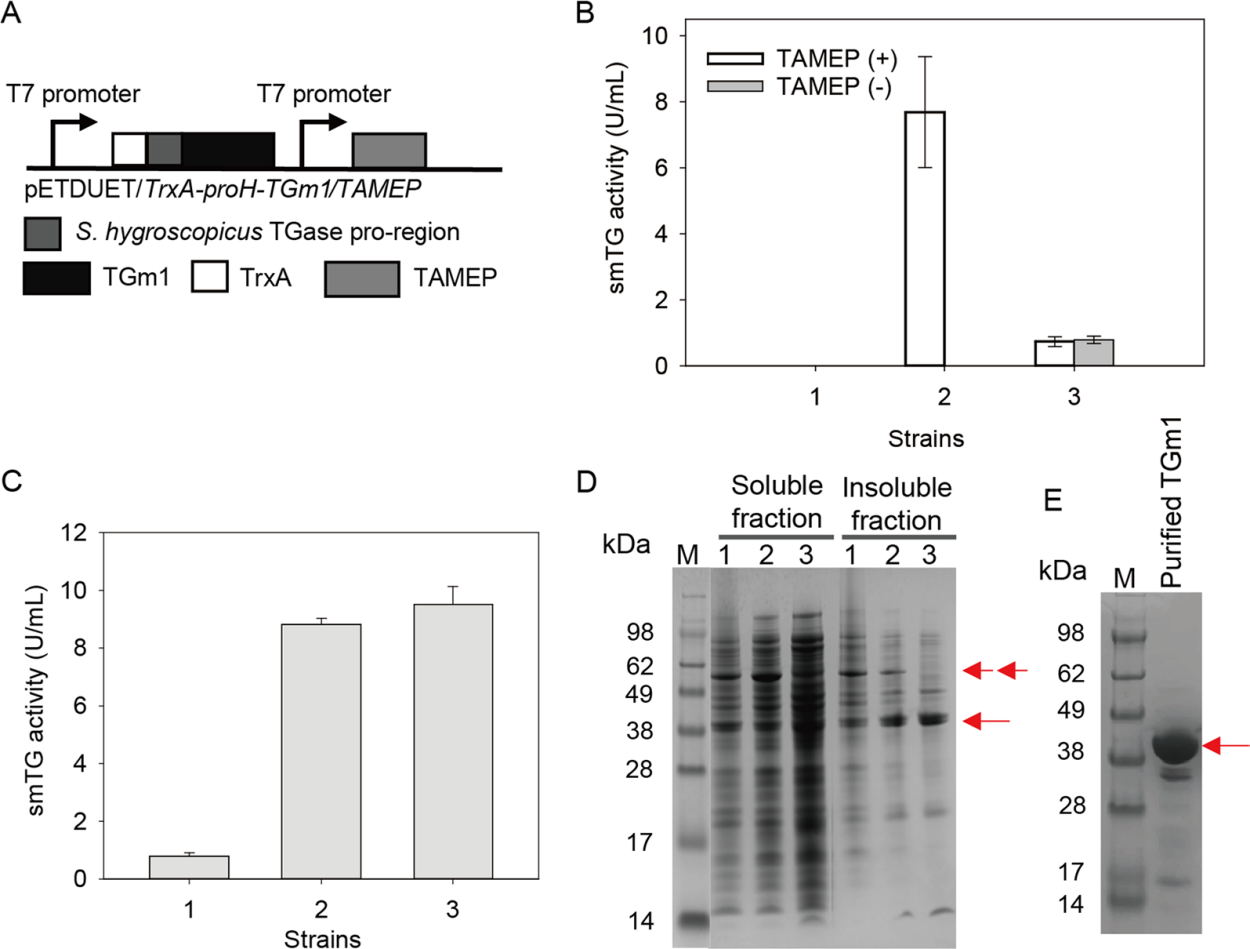
The effect of co-expressing TAMEP and chaperones on the active expression of TGm1 in *E. coli*. **A** The expression cassette for co-expressing TrxA-proH-TGm1 and TAMEP;** B** The intracellular smTG activity of the *E*. *coli* strains with or without co-expressing TAMEP. 1. *E*. *coli* expressing pETDuet-1; 2: *E*. *coli* carrying pETDuet-1/*TrxA-proH-TGm1*; 3: *E*. *coli* carrying pETDuet-1/*TrxA-proH-TGm1/TAMEP*. **C** The intracellular smTG activity and **D** SDS-PAGE analysis of the *E*. *coli* strains with or without co-expressing chaperones. 1: *E*. *coli* carrying pETDuet-1/*TrxA-proH-TGm1/TAMEP*; 2: *E*. *coli* carrying pETDuet-1/*TrxA-proH-TGm1/TAMEP* and pTf16; 3: *E*. *coli* carrying pETDuet-1/*TrxA-proH-TGm1/TAMEP* and pKJE7. Single and double red arrows indicate the positions of mature TGm1 and TrxA-proH-TGm1 bands, respectively. **E** SDS-PAGE analysis of the TGm1 purified from the intracellular fraction of *E*. *coli* carrying pETDuet/*TrxA*-*proH*-*TGm1*/*TAMEP* and pKJE7 using Nickel affinity chromatography. Each recombinant *E. coli* was cultivated in TB medium at 20 ℃ for 32 h after 0.1 mM IPTG addition

Previous result showed that dispase and TAMEP are more efficient for cleaving the pro-region of smTG without degradation of the activated smTG compared to other proteases, such as chymotrypsin, trypsin, and proteinase K [8, 11]. To induce the in vivo TGm1 activation, we constructed the plasmid pETDuet-1/*TrxA-proH-TGm1/TAMEP* that co-expressed TAMEP with TrxA-proH-TGm1 using pETDuet-1 (Fig. 2A). The pETDuet-1/*TrxA-proH-TGm1* was constructed as a negative control solely expressing TrxA-proH-TGm1. As shown in Fig. 2B, the *E. coli* strain carrying pETDuet-1/*TrxA-proH-TGm1/TAMEP* can directly produce 0.79 U/mL of active TGm1, while solely expressing TrxA-proH-TGm1 based on pETDuet-1 showed 7.69 U/mL of the enzyme after the in vitro activation by dispase (Fig. 2B). These results suggest that co-expressing TAMEP could produce active TGm1 but may be detrimental to the TrxA-proH-TGm1 expression.

Molecular chaperones are suggested to promote protein expression by preventing their misfolding [25]. To improve the yield of active TGm1, the chaperone plasmids pTf16 (encoding Triger factor) and pKJE7 (encoding DnaK/DnaJ/GrpE) were transformed into the *E. coli* strain co-expressing TrxA-proH-TGm1 and TAMEP, respectively. As shown in Fig. 2C, co-expressing Triger factor and DnaK/DnaJ/GrpE achieved 8.59 U/mL and 9.51 U/mL of intracellular TGm1, 9.87-fold and 11.03-fold higher than the strain solely expressing TrxA-proH-TGm1 and TAMEP. SDS-PAGE analysis indicated that protein bands slightly bigger than the theoretical molecular size of TGm1 (38 kDa) were observed in both soluble and insoluble fraction of each recombinant strain (Fig. 2D). To be noted, the band close to the theoretical molecular size of TrxA-proH-TGm1 (56.5 kDa) in insoluble fraction of *E. coli* transformed with pETDuet/*TrxA*-*proH*-*TGm1*/*TAMEP* get thinner or even eliminated in the corresponding fractions of the *E. coli* co-expressing chaperones (Fig. 2D). This result suggests that co-expressing the Triger factor or DnaK/DnaJ/GrpE could benefit the soluble expression of TrxA-proH-TGm1. Then, the intracellular soluble fraction of *E. coli* carrying pETDuet/*TrxA*-*proH*-*TGm1* /*TAMEP* and pKJE7 was subjected to the affinity purification. As indicated by SDS-PAGE analysis, TGm1 (38 kDa) dominate the protein composition of the elutes with smTG activity, and no *TrxA*-*proH*-*TGm1* band (56.5 kDa) was detected (Fig. 2E). Moreover, the addition of dispase did not increase the smTG activity of the intracellular soluble fraction of the strain co-expressing pKJE7 (data not shown). Therefore, the additional soluble pro-enzyme induced by the over-expressed chaperone could be completely converted into active enzyme.

Previously, we co-expressed the *S. hygroscopicus* transglutaminase with its native pro-region in *E. coli*, yielding 0.13 U/mL/OD_600_ of the enzyme [9]. This enzyme yield is comparable to the level of *E. coli* co-expressing the proteases and the pro-enzyme inserted with the corresponding cleavage site [12, 13]. By co-expressing molecular chaperones as well as TrxA-proH-TGm1 and TAMEP, the intracellular enzyme activity here reached 9.51 U/mL (equivalent to 2.38 U/mL/OD_600_), significantly higher than the previous reports.

### Improving the secretory expression of active TGm1 by optimizing signal peptide and deleting *lpp*

**Fig. 3:**
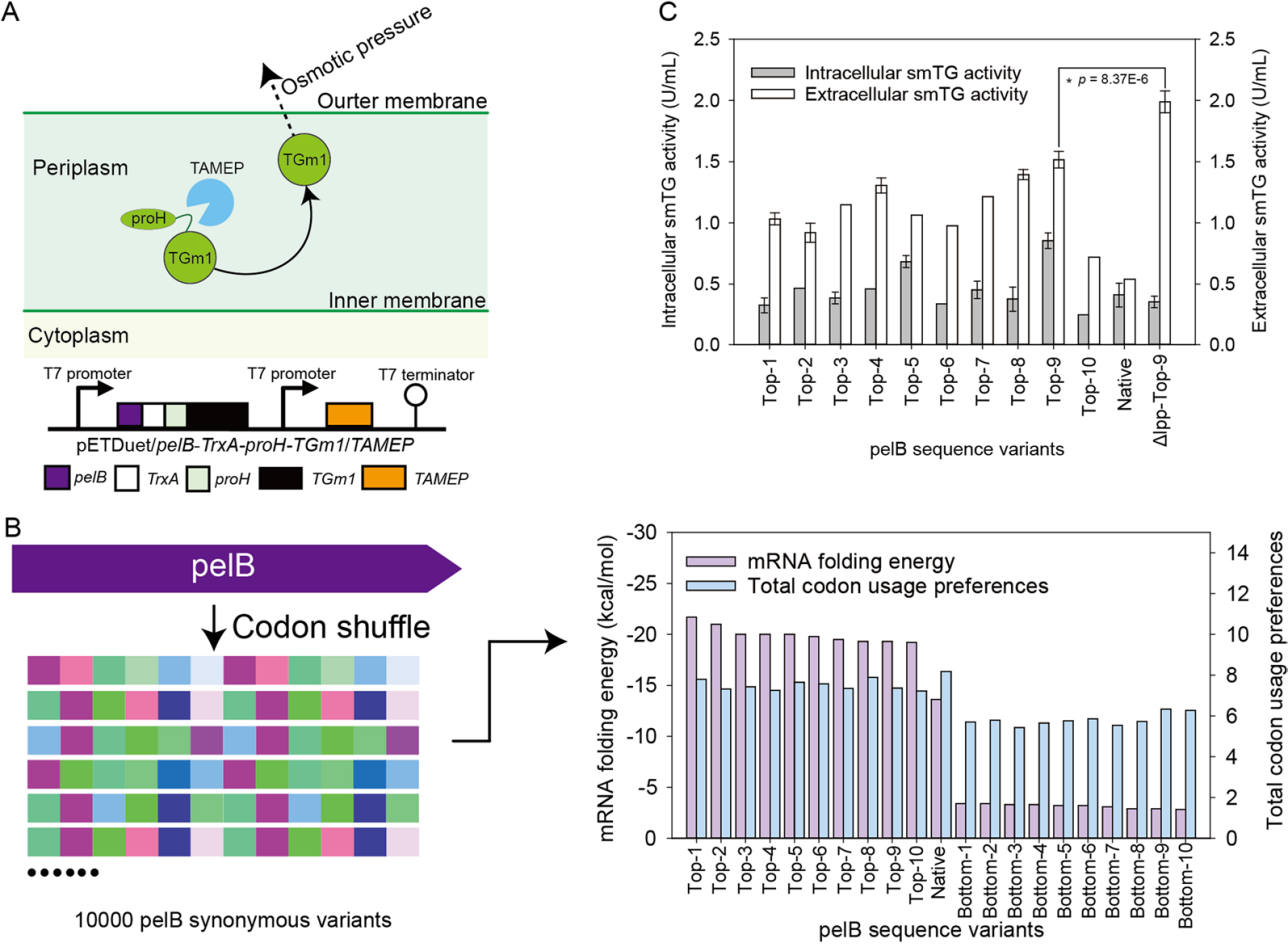
Engineering secretory expression of TGm1 in *E. coli*. **A** Engineering the periplasmic activation of TGm1; **B** The lowest (Top) and highest (Bottom) 10 mRNA folding energy pelB sequence variants achieved by codon shuffling. **C** The intracellular and extracellular activity of TGm1 fused with different pelB sequence variants. Statistically significant differences were determined using Excel Variance Analysis, and the *p* value comparison between Top-9 and Δlpp-Top-9 is displayed and indicated with an asterisk. Native: TGm1 fused with the pelB from pET-22b (+). Δlpp-Top-9: TGm1 fused with the Top-9 pelB, and the plasmid was transformed into *E. coli Δlpp*. Each recombinant *E. coli* was cultivated in TB medium at 20 ℃ for 40 h after 0.1 mM IPTG addition

Excessive intracellular accumulation of smTG may be toxic to *E. coli* [13]. PelB signal peptide is widely used to direct the exportation of heterogeneous proteins into the periplasmic space of *E. coli* [26, 27]. To alleviate the cell stress, we fused pelB to the N-terminus of TrxA-proH-TGm1 by constructing the pETDuet/*pelB*-*TrxA*-*proH*-*TGm1*/*TAMEP*. Because TAMEP bearing a native signal peptide [11], the TrxA-proH-TGm1 thus could be activated by TAMEP in the periplasmic space and diffused into the culture medium via osmotic pressure (Fig. 3A) [26]. After 0.1 mM IPTG induction at 20 ℃ for 40 h, the extracellular and intracellular smTG activities of the *E. coli* carrying pETDuet/*pelB*-*TrxA*-*proH*-*TGm1*/*TAMEP* and pKJE7 reached 0.54 U/mL (Additional file 1: Fig. S4) and 0.41 U/mL (Fig. 3C), respectively. The total activity is much lower than that of the strain expressing pETDuet/*TrxA-proH-TGm1*/TAMEP and pKJE7, suggesting that fusion with the pelB signal peptide inhibits the expression of TrxA-proH-TGm1.

It has been reported that the codon usage of the N-terminal coding sequence significantly affected the enzyme expression in *E. coli* via regulating its mRNA secondary structure [28]. In our previous study, synonymous mutation within N-terminal signal peptides induced remarkable changes in enzyme secretion [29]. To generate the N-terminal pelB synonymous variants varying in mRNA folding energy (Fig. 3B), we developed the script that enables the automatic data processing, including codon shuffling, mRNA folding energy calculating, and selecting the samples with the highest or lowest mRNA folding energy (Additional file 1: Table S1). According to the mRNA folding energy, the Top 10 and Bottom 10 variants (Additional file 1: Table S2) were selected from 10,000 pelB sequence variants (Additional file 1). The Top 10 pelB sequence variants displayed an average mRNA folding energy of − 19.98 kcal/mol, 5.4 and 0.47-fold lower than that of Bottom 10 variants and the native pelB, respectively (Fig. 3B). Then, based on pETDuet/*pelB-TrxA-proH-TGm1*/*TAMEP*, the plasmids encoding TrxA-proH-TGm1 fused the Top 10 and bottom 10 pelB sequence variants were constructed and tested for TGm1 production in presence of pKJE7. Compared to the native pelB, all of the Top 10 pelB sequence variants achieved relatively higher extracellular smTG activities, and latter also induced more intracellular enzyme except Top-5 and Top-9 variants (Fig. 3C). Among them, Top-9 variant obtained 1.52 U/mL of extracellular enzyme activity, 1.8-fold higher than that generated by the native signal peptide (Fig. 3C). In contrast, we did not detect intra- or extracellular smTG activity in the case of the Bottom 10 pelB sequence variants. To be noted, the native pelB sequence showed a higher *E. coli* codon preference compared to the Top and Bottom 10 pelB sequence variants (Fig. 3B), suggesting that the codon usage itself does not dominate the enzyme expression. Instead, strong mRNA folding (low mRNA folding energy) within the pelB signal peptide may account for the expression of TGm1 in *E. coli*. It has demonstrated that strong mRNA folding between the first 13–40 codons of proteins might be favorable for ribosomal allocation and reduce ribosome traffic jams during “late translation initiation” the N-terminal coding sequence [30]. The gene of pelB signal peptide is composed of the first 21 codons that intersect the functional regions (Additional file 1: Table S2). Thus, there is a possibility that the Top 10 pelB variants with the improved enzyme production shared the similar mechanism.

The *lpp* is suggested to be responsible for the outer membrane permeability of *E. coli* [21]. Thus, *lpp* was deleted in the recombinant strain with Top-9 pelB variant to further enhance the extracellular expression of TGm1. As shown in Fig. 3C, the smTG activity secreted by the *lpp* deletion strain reached 1.99 U/mL, 31.2% higher than that in the control strain; in contrast, extracellular smTG activity was decreased by 58.6%; variance analysis indicated that the *lpp* deletion led to a significant increase (*p* = 8.37E−6) in extracellular expression of TGm1 in *E. coli*. To be noted, the overall smTG activity of the native (2.37 U/mL) was slightly different from that of and *Δlpp* strain (2.34 U/mL) (Fig. 3C), suggesting that deleting *lpp* mainly affected the enzyme exportation instead of the expression. In previous studies, we achieved 40 U/mL of smTG in *Streptomyces* by physical mutagenesis and rational design [2]. With the expansion of the application fields [31], the low catalytic activity, narrow substrate specificity, and weak thermal stability gradually became the main obstacles for the application of the wild-type smTG. However, the low growth rate and difficulty in genetic manipulation prevented *Streptomyces* being an ideal platform for smTG modification. After achieving the intracellular expression of smTG in *E. coli*, we improved the thermostability and catalytic efficiency of the enzyme by rational design [32]. Because extracellular expression is more favorable for enzyme isolation and activity determination, the *∆lpp E. coli* strain constructed here is thus more applicable as a high throughput screening platform for evolving smTG.

### Characterizing the thermostability and organic solvent tolerance of FRAPD-TGm1

**Fig. 4:**
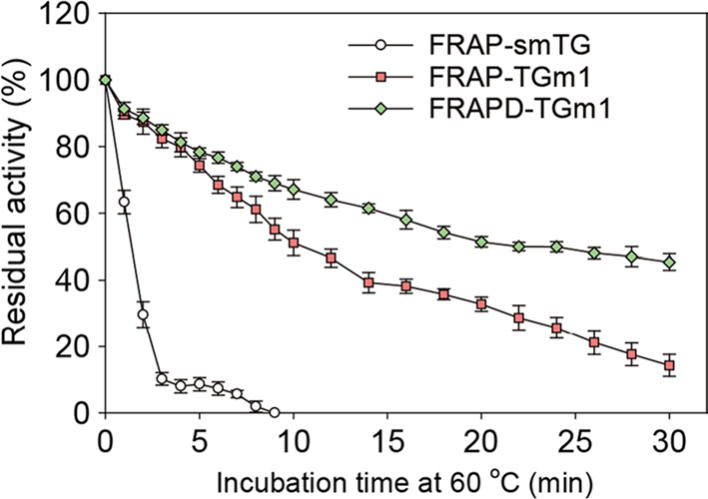
The activation cure of smTG and its variants. Residual activity of smTG variants under 60 ℃ incubation. FRAP-smTG and its variants were expressed in *E. coli*, which carrying plasmid pET-22b/*pro*-*smTG*, pET-22b/pro-*TGm1*, and pETDuet-1/*TrxA-proH-TGm1*/*TAMEP*. The fermentation temperature was under 20 ℃ after induction by adding IPTG at a final concentration of 0.1 mM. Recombinant proteins were purified by affinity purification using the His-Trap column and SEC, which finally eluted in Tris–HCl (50 mM, pH 8.0)

As mentioned, the activation of proH-TGm1 by dispase yielded FRAPD-TGm1, which has an additional Asp compared to the FRAP-TGm1 generated from proH-TGm1 using the same activating enzyme (Additional file 1: Fig. S3). In the present study, we investigated the catalytic properties of FRAP-TGm1 and FRAPD-TGm1. As shown in Fig. 4, FRAPD-TGm1 was more stable than FRAP-TGm1 when treated with water bath at 60 ℃. Compared to FRAP-TGm1, the melting temperature and half-life at 60 ℃ of FRAPD-TGm1 were increased by 0.95 ℃ and 94.3%, respectively (Table 1). In contrast, FRAPD-TGm1 shared similar specific activity with FRAP-TGm1 (Table 1). These results suggested that the additional Asp residue improved the thermostability of FRAPD-TGm1 rather than the enzyme activity. In addition to the thermostability, the organic solvent tolerance of transglutaminase is also critical for crosslinking peptides or small molecules which are only soluble in aqueous solution [33]. In the present study, we examined the activities of FRAP-TGm1 and FRAPD-TGm1 in the solutions containing 10–30% of DMSO, ethanol, DMF, or Methanol, respectively. The relative activity in each organic solvent solution is normalized to the optimal buffer, where the activity of each TGm1 variant in the optimal buffer is expressed as 100%. As shown in Table 2, both FRAP-TGm1 and FRAPD-TGm1 displayed over 45% of the relative activity in the solutions containing 30% of each tested organic solvent. When tested in 10–25% DMSO or 10–30% DMF, FRAP-TGm1 and FRAPD-TGm1 shared similar enzymatic activities (Table 2). However, compared to FRAPD-TGm1 was less active in 30% DMSO. In all the tested concentration cases, FRAPD-TGm1 displayed higher activities in ethanol or methanol solutions compared to FRAP-TGm1 (Table 2). Thus, FRAPD-TGm1 may be a robust candidate for crosslinking those alcohol-soluble substrates, while FRAPD-TGm1 exhibit a great potential in the reaction using DMSO as the solvent, such as peptide synthesis [33].

**Table 1:** The thermostabilities and specific activities of smTG variants

Parameters	FRAP-smTG	FRAP-TGm1	FRAPD-TGm1
Specific activity (U/mg)	26	50	49.14
*t*_1/2_^60 ℃^ (min)	< 2	11.31	21.97
*T*_m_ (℃)	56.09	64.22	65.17

**Table 2:** Organic solvent tolerance of FRAP-TGm1 and FRAPD-TGm1

Solvent type	Solvent concentration (v/v) (%)	Relative activity (%)	
		FRAP-TGm1	FRAPD-TGm1
DMSO	10	87.9 ± + 2.44	84.8 ± 0.71
	15	79.8 ± 1.73	79.1 ± 2.54
	20	70.7 ± 2.00	74.8 ± 2.46
	25	63.8 ± 2.66	65.7 ± 2.66
	30	59.3 ± 2.25	47.4 ± 1.44
Ethanol	10	71.2 ± 0.80	75.2 ± 1.56
	15	67.9 ± 1.35	71.5 ± 2.83
	20	63.2 ± 1.14	67.2 ± 0.60
	25	59.3 ± 1.38	65.2 ± 2.89
	30	53.0 ± 0.62	60.0 ± 1.80
DMF	10	70.1 ± 3.96	67.3 ± 1.68
	15	64.0 + 2.22	64.2 ± 1.55
	20	58.3 ± 3.54	62.4 ± 2.12
	25	53.4 ± 2.38	53.6 ± 1.08
	30	50.3 ± 2.71	48.6 ± 3.56
Methanol	10	86.8 ± 1.32	92.0 ± 3.71
	15	84.0 ± 1.56	90.2 ± 2.59
	20	83.9 ± 2.50	87.9 ± 1.58
	25	81.9 ± 1.71	86.1 ± 1.19
	30	77.4 ± 3.11	83.3 ± 1.67

### Understand the stabilization mechanism of FRAPD-TGm1

**Fig. 5:**
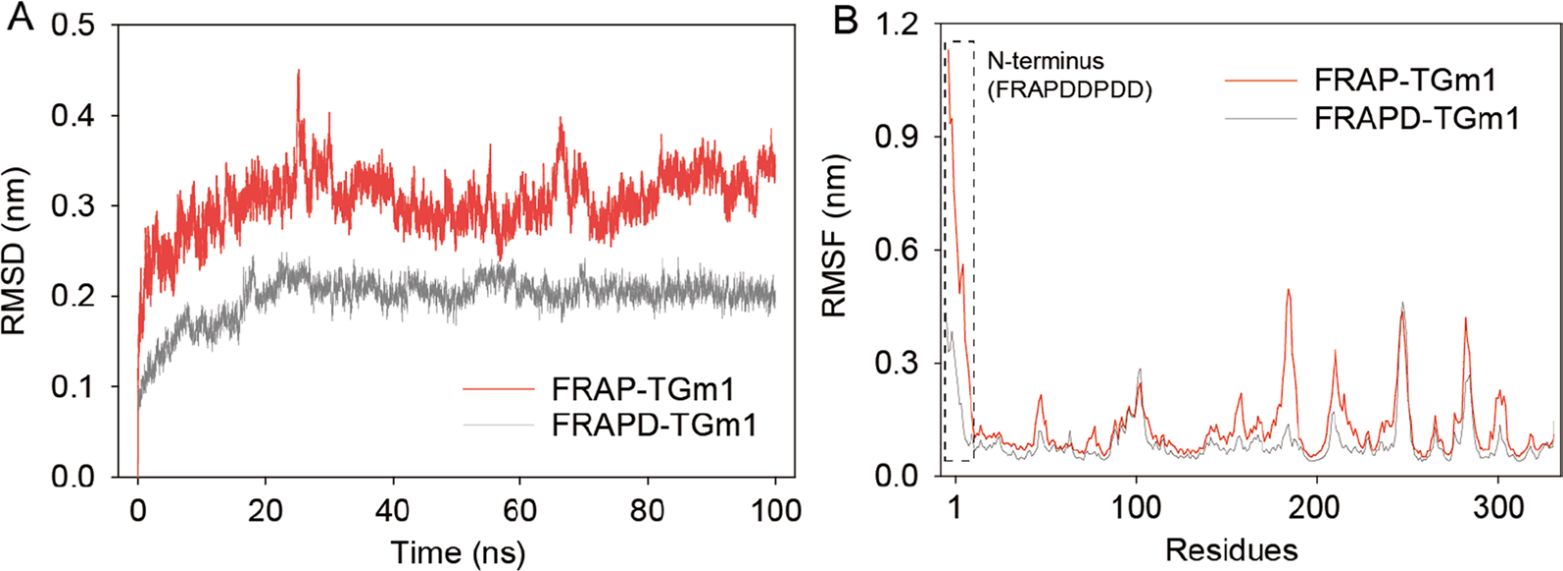
Molecular dynamics simulation of smTG variants. A RMSD analysis, **B** RMSF analysis. The structure of FRAP-TGm1 and FRAPD-TGm1 were modelled by I-TASSER, and subjected to MD simulation using GROMACS-2020 at 330 K

To understand the mechanism behind increased thermostability, we modeled the structures of FRAP-TGm1 and FRAPD-TGm1 and conducted MD simulation. The system showed a stabilized trend after 30 ns simulation, and the average RMSD of FRAPD-TGm1 (0.198) was much lower than that of FRAP-TGm1 (0.305) at 330 K (Fig. 5A). Generally, the rigidity of an enzyme is positively related to its stability [32]. Thus, the improved thermostability of FRAPD-TGm1 may be due to the increased structural rigidity. As shown in Fig. 5B, we observed a significant RMSF decline in N-terminus of FRAPD-TGm1 (FRAPDDPDD). During MD analysis, the average hydrogen bond formation frequencies within the N-terminal region of FRAPD-TGm1 and FRAP-TGm1 were 3.49 and 1.57, respectively (Additional file 1: Figs. S5A and S7B). The N-terminus of TGm1 is a long and flexible loop structure, consisting of 31 amino acids (including the FRAP tag) (Additional file 1: Fig. S5C). It has demonstrated that hyper flexible areas were much easier to trigger protein denaturation in an early stage [34]. Therefore, the additional Asp residue induced the N-terminal flexibility through hydrogen bond formation, which might inhibit protein denaturation of FRAPD-TGm1 in an early stage.

### Conclusions

In this study, we explored methods to overexpress TGm1 in *E. coli*, and simultaneously generated a thermostable TGm1 variant FRAPD-TGm1 as a result of removing the pro-region of proH-TGm1 in vitro. We subsequently investigated strategies for overexpressing active TGm1 in *E. coli* by co-expressing TAMEP and TrxA-proH-TGm1, with chaperones DnaK/DnaJ/GrpE. To achieve extracellular active expression of TGm1, signal peptide pelB was fused to TrxA-proH-TGm1. Codon optimization of pelB was carried out according to the mRNA folding energy, which revealed that pelB sequence variants with lower mRNA folding energy can promote the expression and secretion of TGm1. In addition, the extracellular expression of TGm1 was enhanced by using Top9 pelB variant in the ∆*lpp E. coli* strain.

We characterized the organic solvent tolerance of FRAP-TGm1 and FRAPD-TGm1, and found that the two variants maintained high activity at the presence of 30% organic solvent in the substrate solution. MD simulation revealed that the increased thermostability of FRAPD-TGm1 may as a result of the additional Asp at the N-terminus can enhance the local rigidity. The thermostable TGm1 variant can be a robust candidate for smTG involved applications, as well as a further modification target. The platform for secretory expression of active TGm1 in *E. coli* can be used for producing TGm1 or high-throughput screening to evolve TGm1.

## Materials and methods

### Materials

All general chemicals and lab reagents were purchased in AR grade from Sangon Biotech (Shanghai, China) unless otherwise specified. CBZ-Gln-Gly was purchased from Sigma-Aldrich (Shanghai, China), and dispase was purchased from Solarbio, Beijing, China.

### Strains and plasmids

All plasmids were listed in Additional file 1: Table S3. Since all plasmids were circularized in vitro, we directly used *E*. *coli* BL21 (DE3) as the cloning and protein expression strain.

### Plasmid construction

The gene encoding for pro-TGm1 (GenBank ID: MZ516369, Additional file 1: Fig. S6) and TAMEP (GenBank ID: MZ516816, Additional file 1: Fig. S7) were optimized for *E*. *coli* expression, synthesized and cloned into pET-22b and pETDuet-1 via *Nde*I and *Blp*I sites, yielding the plasmids pET-22b/*pro*-*TGm1* and pETDuet/*TAMEP*, respectively (GENEWIZ, Suzhou, China). The gene encoding the pro-regions of different *Streptomyces* were synthesized as the reference study [14] (Additional file 1: Table S4). Construction of plasmids expressing TGm1 with different pro-regions was carried out by two steps. Firstly, a vector containing homologous arm with different pro-regions was generated by PCR using ptgvf and ptgvr primers, with plasmid pET-22b/*pro*-*TGm1* as template (Additional file 1: Table S5). Secondly, the synthesized pro-regions were ligated with the vector using ClonExpress II One Step Cloning Kit (Vazyme, Nanjing, China) (Additional file 1: Table S5).

The gene encoding TrxA (GenBank ID: KJ183182.1) was achieved by PCR using ptrxf and ptrxr as primers, with plasmid pET-48b (+) as template (Additional file 1: Table S5). The gene encoding MBP achieved by PCR using the genome of *E*. *coli* BL21 (DE3) as template, and pmbpf and pmbpr as primers (Additional file 1: Table S5). A vector for *TrxA* and *MBP* insertion was generated by PCR using pET-22b/*proH*-*TGm1* as template, and ptmvf and ptmvr as primers (Additional file 1: Table S5). *TrxA* and *MBP* were ligated with the vector using ClonExpress II One Step Cloning Kit (Vazyme, Nanjing, China) to obtain pET-22b/*TrxA*-*proH*-*TGm1* and pET-22b/*MBP*-*proH*-*TGm1*, respectively.

The vector for *TrxA*-*proH*-*TGm1* insertion was generated by PCR using pthtvf and pthtvf as primers, with plasmid pETDuet/*TAMEP* as template (Additional file 1: Table S5). *TrxA*-*proH*-*TGm1* was amplified using pET-22b/*TrxA*-*proH*-*TGm1* as template, and pthtf and pthtr as primers (Additional file 1: Table S5). *TrxA*-*proH*-*TGm1* was ligated with the vector using ClonExpress II One Step Cloning Kit (Vazyme, Nanjing, China) to obtain pETDuet/*TrxA*-*proH*-*TGm1*/*TAMEP*. *TAMEP* was further removed from the plasmid pETDuet/*TrxA*-*proH*-*TGm1*/*TAMEP* by PCR using ptampf and ptampr as primers (Additional file 1: Table S5).

pETDuet/*pelb*-*TrxA*-*proH*-*TGm1*/*TAMEP* carrying the gene encoding for pelB sequence variants was generated by PCR using the forward (Top1f-Top10f, and Bot1f-Bot10f) and reverse (Top1r-Top10r, and Bot1r-Bot10r) primers listed in Additional file 1: Table S5, with plasmid pETDuet/*TrxA*-*proH*-*TGm1*/*TAMEP* as template The PCR product was circularized by Blunting Kination Ligation Kit (TaKaRa, Dalian, China). For knockout *lpp* in *E. coli*, pTargetF-*lpp* encoding the sgRNA for cleaving *lpp* site was also constructed using PCR, and ligated with ClonExpress II One Step Cloning Kit (Additional file 1: Table S5).

### Construction of *lpp* deficient *E*. *coli* BL21 (DE3) strain

Plasmid pCas was transformed into *E*. *coli* BL21 (DE3) by chemical transformation [35]. The upstream and downstream DNA flanked *lpp* were achieved by PCR using *E*. *coli* genome as template and annealed. pTargetF and the annealed DNA were co-transformed into *E*. *coli* carrying pCas by electroporation [36]. pCas and pTargetF were separately removed through incubating *E*. *coli* under 40 ℃ and induction by adding IPTG [36]. Knockout of *lpp* was verified by colony PCR (Additional file 1: Table S5) and gene sequencing.

### Protein expression

The plasmid encoding for TAMEP, and TGm1 with different pro-regions and fusion proteins were chemical transformed into *E*. *coli* BL21 (DE3) [35]. Single colony of obtained from *E*. *coli* carrying different plasmids was inoculated into Luria–Bertani (LB) medium supplemented with 50 µg/mL ampicillin for seed culture at 37 ℃ for 10 h. The seed culture was transferred into Terrifc-broth (TB) supplemented with 50 µg/mL ampicillin and cultivated until the cell density (OD_600_) reached to 1.0. IPTG was subsequently supplemented to 0.1 mM for recombinant expressing smTG and TAMEP, and cells cultivation was under 20 ℃. For IPTG induced TrxA-proH-TGm1 and TAMEP co-expression, IPTG was supplemented to a final concentration of 0.1 mM while OD_600_ reached to 1.0, and cells were continuously cultivated at 20 ℃.

### Protein purification

Cells were obtained from the fermentation culture by centrifugation, and resuspended in Tris–HCl (50 mM, pH 8.0) for ultra-sonification. The pro-region of smTG variants was removed using dispase at a final concentration of 2 mg/mL and incubated at 37 ℃ for 30 min. The supernatant contained TGm1 variants was subjected to affinity purification using the His-Trap column (GE Healthcare, New York, USA) and size-exclusion chromatography (SEC) using Superdex 75 column (GE Healthcare, New York, USA), and finally eluted in Tris–HCl (50 mM, pH 8.0) for enzyme analysis. TAMEP was purified by affinity purification and SEC as is shown above, and finally eluted in 50 mM Tris/HCl buffer, 100 mM NaCl, 2 mM CaCl_2_, 2 mM GSH, pH 8.0.

### Thermostability analysis

The purified TGm1 variants were adjusted to 0.4 mg/mL for measuring the residual activity and characterizing the *t*_1/2_. Samples were treated at 60 ℃ water bath and a portion were taken out at specific intervals for testing. After thermal incubation, the samples were cooled down on ice and centrifuged to remove the precipitates before smTG activity assay. The *t*_1/2_ of each enzyme was calculated using Origin 2019 exponential fit. The *T*_m_ of smTG variants were determined by differential scanning calorimeter using differential scanning calorimeter (Nano-DSC, TA instruments, New Castle, USA). The system pressure was set to 3 atmospheres, and the temperature raised from 40 to 90 ℃ by 1 ℃ per min that enthalpy changes data were used for *T*_m_ calculation.

### Protein analysis

Protein concentration was measured by Bradford Protein Assay Kit (Beyotime, Shanghai, China), and the N-terminal amino acid sequencing was conducted using Edman degradation method (BiotechPack Scientific, Beijing, China). SDS-PAGE was performed using 12% Tris–glycine gel (ThermoFisher, Shanghai, China).

### Determination of transglutaminase activity

To measure the specific activity of smTG, 60 μL of pre-warmed (at 37 ℃ for 5 min) sample protein was added to 150 μL substrate solution (Tris–HCl 200 mM, 100 mM hydroxylamine, 10 mM GSH, 30 mM CBZ-Gln-Gly, pH 6.0), and the reaction last for 10 min under 37 ℃ which terminated by 60 μL of termination solution (termination solution achieved by mixing same volume of 3 M HCl, 12% trichloroacetic acid and 5% FeCl_3_·H_2_O) [37]. For measuring smTG organic solvent tolerance, the substrate solution was supplemented with 10–30% of organic solvent, including DMF, DMSO, ethanol, and methanol. One unit of smTG activity was defined as 1 μmol l-glutamic acid γ-monohydroxamate produced per min.

### Bioinformatic and statistical analysis

The structure of FRAP-TGm1 and FRAPD-TGm1 were separately modelled by I-TASSER (https://zhanglab.ccmb.med.umich.edu/I-TASSER/), and energy minimized by Rosetta relax module [38, 39]. MD simulation was performed using GROMACS-2020 (Uppsala University, Uppsala, Sweden), and TGm1 variants were embedded with FF14sb force field [40, 41]. The structures were solvated in SPC/E water within a cubic box of 12 Å to the horizon. The system was neutralized by Na^+^ and Cl^−^, and energy minimized by steepest descent method followed by isochoric-isothermal ensemble and isothermal-isovolumetric ensemble for energy equivalent under 330 K. The simulation was carried out for 100 ns under 330 K, and the trajectories were analyzed. A pelB codon shuffling script was developed, and we achieved 100,000 pelB synonymous variants (Additional file 1: Table S1). Secondary structure prediction and the lowest energy evaluation of the generated 100,000 sequences were conducted by RNAstructure fold module [42]. The statistically significant differences were performed using Excel Variance Analysis to compare group variances.

## Supplementary Information

**Additional file 1: Table S1. **Scripts used in this study. **Table S2.** mRNA folding energy based Top 10 and Bottom 10 variants. **Table S3. **Plasmids used in this study. **Table S4. **Synthetic double strand DNA fragment used in this study. **Table S5. **Primers used in this study. **Figure S1. **Expression of TGm1 in different forms. *E. coli *transformed with **A** pET-22b/pro-TGm1, **B** pET-22b/proH-TGm1, and **C** pET-22b-trxA-proH-TGm1. 1, 3, and 5: insoluble fractions; 2, 4, and 6: soluble fractions. *E. coli* was cultivated at 20 ℃ for 32 h after induction by adding IPTG at a final concentration of 0.1 mM when OD_600_ reached 1.0. Red arrow: **A** pro-TGm1, **B** proH-TGm1, and **C** TrxA-proH-TGm1. **Figure S2.** SDS-PAGE analysis of smTG variants purified by Nickel affinity chromatography. 1: FRAP-TGm1; 2: FRAPD-TGm1. **Figure S3.** Edman sequencing the N-terminus of recombinant TGm1 variants activated by dispase *in vitro*. pro-TGm1 and proH-TGm1 were intracellularly expressed in *E*. *coli* using pET-22b/*pro-TGm1* and pET-22b/*proH-TGm1*, recombinantly expressed TGm1 variants were *in vitro* activated and purified by affinity chromatography using HisTrap (GE Healthcare, USA) column. **Figure S4.** *E. coli* carrying pETDuet/*pelB*-*TrxA*-*proH*-*TGm1*/*TAMEP *and pKJE7 was cultivated under 20 ℃ after induction by IPTG. **Figure S5.** MD simulation of FRAP-TGm1 and FRAPD-TGm1. **A** Hydrogen bonds within the N-terminus (FRAPDPDD) of FRAP-TGm1. **B** Hydrogen bonds within the N-terminus (FRAPDPDD) of FRAPD-TGm1. MD simulation was conducted for 100 ns using Gromacs-2020. **C** The modeled structure of FRAPD-TGm1. The N-terminal loop of FRAPD-TGm1 was colored in cyan. **Figure S6.** Sequence analysis of pro-TGm1 (GenBank ID: MZ516369). The number of nucleotide sequences (left side); Single underline: *pro-region*; Double underline: mature region of *TGm1*; Box, His-tag. **Figure S7.** Sequence analysis of TAMEP (GenBank ID: MZ516816). A start codon (ATG) was placed after the predicted signal peptide. Single underline: signal peptide [2]. Numbers (left side): the number of nucleotide sequences. Box, His-tag. For purification purpose, a His-tag was placed as shown in box while constructing the plasmid pETDuet/TAMEP; for co-expressing TAMEP with TGm1, the His-tag (as shown in box) was removed.

## Data Availability

All data generated or analyzed during this study are included in this published article and its Additional file 1.

## Abbreviations

MBP: Maltose-binding protein
TrxA: Thioredoxin-1
PCR: Polymerase chain reaction
DMF: Dimethylformamide
DMSO: Dimethyl sulfoxide
MD: Molecular dynamics
CBZ-Gln-Gly: N-carbobenzoxy (CBZ)-l-glutaminylglycine-Gln-Gly
AR: The standard Mallinckrodt grade of analytical reagents
*F crit*: Critical value
*F*: The value of the test statistic
*p*: Probability value
