# Supplementary material for: Active secretion of a thermostable transglutaminase variant in Escherichia coli
Source: Microb Cell Fact. 2022 Apr 29;21:74. doi: 10.1186/s12934-022-01801-9 (PMC9052465; doi:10.1186/s12934-022-01801-9)
Supplement: Supplementary file 1 — Additional file 1: Table S1. Scripts used in this study. Table S2. mRNA folding energy based Top 10 and Bottom 10 variants. Table S3. Plasmids used in this study. Table S4. Synthetic double strand DNA fragment used in this study. Table S5. Primers used in this study. Figure S1. Expression of TGm1 in different forms. E. coli transformed with A pET-22b/pro-TGm1, B pET-22b/proH-TGm1, and C pET-22b-trxA-proH-TGm1. 1, 3, and 5: insoluble fractions; 2, 4, and 6: soluble fractions. E. coli was cultivated at 20 ℃ for 32 h after induction by adding IPTG at a final concentration of 0.1 mM when OD600 reached 1.0. Red arrow: A pro-TGm1, B proH-TGm1, and C TrxA-proH-TGm1. Figure S2. SDS-PAGE analysis of smTG variants purified by Nickel affinity chromatography. 1: FRAP-TGm1; 2: FRAPD-TGm1. Figure S3. Edman sequencing the N-terminus of recombinant TGm1 variants activated by dispase in vitro. pro-TGm1 and proH-TGm1 were intracellularly expressed in E. coli using pET-22b/pro-TGm1 and pET-22b/proH-TGm1, recombinantly expressed TGm1 variants were in vitro activated and purified by affinity chromatography using HisTrap (GE Healthcare, USA) column. Figure S4. E. coli carrying pETDuet/pelB-TrxA-proH-TGm1/TAMEP and pKJE7 was cultivated under 20 ℃ after induction by IPTG. Figure S5. MD simulation of FRAP-TGm1 and FRAPD-TGm1. A Hydrogen bonds within the N-terminus (FRAPDPDD) of FRAP-TGm1. B Hydrogen bonds within the N-terminus (FRAPDPDD) of FRAPD-TGm1. MD simulation was conducted for 100 ns using Gromacs-2020. C The modeled structure of FRAPD-TGm1. The N-terminal loop of FRAPD-TGm1 was colored in cyan. Figure S6. Sequence analysis of pro-TGm1 (GenBank ID: MZ516369). The number of nucleotide sequences (left side); Single underline: pro-region; Double underline: mature region of TGm1; Box, His-tag. Figure S7. Sequence analysis of TAMEP (GenBank ID: MZ516816). A start codon (ATG) was placed after the predicted signal peptide. Single underline: signal peptide [2]. Numbers (left side): the numbe [file 12934_2022_1801_MOESM1_ESM.docx]

**Active secretion of a thermostable transglutaminase variant in *Escherichia coli***

Xinglong Wang^1, 2, 4^, Beichen Zhao^1, 2^, Jianhui Du^1, 2^, Yameng Xu^2^, Xuewen Zhu^2^, Jingwen Zhou^1, 2, 4^, Shengqi Rao^5^, Guocheng Du^2, 3^, Jian Chen^1, 2, 4^, Song Liu^1, 2,^ *

1. National Engineering Laboratory for Cereal Fermentation Technology, Jiangnan University, 1800 Lihu Road, Wuxi, Jiangsu 214122, China
2. Science Center for Future Foods, Jiangnan University, 1800 Lihu Road, Wuxi, Jiangsu 214122, China
3. The Key Laboratory of Carbohydrate Chemistry and Biotechnology, Ministry of Education, Jiangnan University, 1800 Lihu Road, Wuxi, Jiangsu 214122, China
4. Jiangsu Provisional Research Center for Bioactive Product Processing Technology, Jiangnan University, 1800 Lihu Road, Wuxi, Jiangsu 214122, China
5. College of Food Science and Engineering, Yangzhou University, Yangzhou, Jiangsu 214122, China

* Corresponding author: Song Liu, Tel: +86-510-85918307, Fax: +86-510-85918309, liusong@jiangnan.edu.cn.

**Tables**

**Table S1** Scripts used in this study.

| **Aims** | **Scripts and parameters** |
| --- | --- |
| Codon shuffling | def Randomcode(path,seq, total_num):  import random  code_dict = {'F':['UUU','UUC'],'L':['UUA','UUG','CUU','CUC','CUA','CUG'],'I':['AUU','AUC','AUA'],'M':['AUG'],'V':['GUU','GUC','GUA','GUG'],'S':['AGU','AGC','UCU','UCC','UCA','UCG'],'P':['CCU','CCC','CCA','CCG'],'T':['ACU','ACC','ACA','ACG'],'A':['GCU','GCC','GCA','GCG'],'Y':['UAU','UAC'],'end':['UAA','UAG','UGA'],'H':['CAU','CAC'],'Q':['CAA','CAG'],'N':['AAU','AAC'],'K':['AAA','AAG'],'D':['GAU','GAC'],'E':['GAA','GAG'],'C':['UGU','UGC'],'W':['UGG'],'R':['CGU','CGC','CGA','CGG','AGA','AGG'],'G':['GGU','GGC','GGA','GGG']}  code_seq = []  for i in range (total_num):  for aa in seq:  aa= random.choice(list(code_dict[aa]))  code_seq=code_seq+[aa]  z=''.join(code_seq)  count=0  for k in range (total_num):  f=open ('%s\\%s.fasta'%(path,k),'w')  f.write(z[(count*(len(seq)*3)) : (len(seq)*3)*(count+1)])  f.close()  count=count+1    #Run the script like of "Randomcode('KYLLPTAAAGLLLLAAQPAMA','E:\\pelB\\all_seq',10000)" to shuffle the codon. |
| Calculate the RNA folding energy | def RNAcal(input_path,output_path,total_num):  import os  for i in range (total_num):  os.system("D:\\interfaceRNAstructure\\RNAstructure6.2\\exe\\Fold.exe %s %s --DNA"%(input_path + '\\' + str(i) + '.fasta',output_path + '\\'+ str(i) + '.ct'))  #Replace the "D:\\interfaceRNAstructure\\RNAstructure6.2\\exe\\Fold.exe" to where your fold function of your RNAstructure is placed.  #Run the script like "RNAcal("E:\\pelB\\all_seq",'E:\\pelB\\output',10000)" to export all the folded RNA structure with its folding energy. |
| Sorting the lowest/highest RNA folding energy samples | def energy_rank(path,total_num):  import pandas as pd  list=[]  for x in range(total_num):  f=open(r'%s\\%s.ct'%(path,x))  info=f.readlines()  count =0  for i in info:  count=count+1  a=i.split()  if count<2:  list.append (a[3])  f.close()  try:  newfile=open('%s\\all_energy.txt'%path,'w')  newfile.close()  for energy in list:  new1=open('%s\\all_energy.txt'%path,'a+')  new1.write(energy+'\n')  new1.close()    except:  for energy in list:  new1=open('%s\\all_energy.txt'%path,'a+')  new1.write(energy+'\n')  new1.close()  print(min(list)) ##sort the lowest RNA folding energy samples  print(max(list)) ##sort the highest RNA folding energy samples  #Run this script like "energy_rank('E:\\pelB\\output',10000)" to sort the lowest RNA folding energy samples. |

**Table S2** mRNA folding energy based Top 10 and Bottom 10 variants

| **Name** | **Sequences (5′-3′)** | **RNA folding energy (kcal/mol)** |
| --- | --- | --- |
| **Top-1** | **AAAUAUUUAUUGCCCACAGCAGCGGCCGGCCUCCUGCUCUUAGCUGCGCAGCCAGCGAUGGCG** | **-21.7** |
| **Top-2** | **AAAUAUCUCCUUCCCACCGCUGCUGCUGGCCUUCUUUUGUUAGCCGCGCAGCCGGCUAUGGCA** | **-21** |
| **Top-3** | **AAAUACCUUUUGCCAACGGCCGCUGCAGGGCUGCUACUUCUUGCAGCCCAACCCGCCAUGGCU** | **-20** |
| **Top-4** | **AAGUAUUUGCUUCCUACUGCAGCGGCCGGACUUUUGCUACUCGCUGCGCAGCCCGCCAUGGCU** | **-20** |
| **Top-5** | **AAGUACCUCCUCCCGACUGCUGCUGCGGGACUACUACUCUUGGCCGCACAACCCGCCAUGGCA** | **-20** |
| **Top-6** | **AAAUACCUACUCCCUACCGCAGCAGCUGGCCUGCUUCUCCUCGCCGCCCAGCCCGCAAUGGCC** | **-19.8** |
| **Top-7** | **AAAUAUCUCCUUCCCACCGCAGCGGCUGGUCUCUUGUUACUCGCCGCCCAGCCAGCAAUGGCG** | **-19.5** |
| **Top-8** | **AAGUAUCUUUUACCGACGGCGGCCGCAGGGCUUUUGCUACUCGCGGCACAGCCUGCUAUGGCU** | **-19.3** |
| **Top-9** | **AAGUAUCUACUACCUACAGCUGCGGCCGGCCUGCUCUUACUAGCAGCUCAGCCGGCGAUGGCA** | **-19.3** |
| **Top-10** | **AAAUACUUACUACCGACGGCGGCGGCCGGCCUGCUCUUGUUGGCUGCACAACCCGCUAUGGCA** | **-19.2** |
| **Bottom-1** | **AAGUAUCUGUUGCCCACCGCCGCCGCCGGGUUGCUACUUUUGGCCGCCCAGCCCGCCAUGGCA** | **-3.4** |
| **Bottom-2** | **AAGUACCUACUGCCUACAGCUGCUGCCGGGUUAUUACUACUUGCUGCGCAGCCAGCAAUGGCA** | **-3.4** |
| **Bottom-3** | **AAGUACCUGCUUCCCACCGCUGCUGCGGGUUUGCUUCUCCUGGCUGCGCAGCCUGCGAUGGCC** | **-3.3** |
| **Bottom-4** | **AAAUACCUGCUACCUACCGCCGCUGCCGGCUUGCUGCUUCUAGCUGCACAGCCGGCCAUGGCU** | **-3.3** |
| **Bottom-5** | **AAAUAUCUCCUCCCCACCGCAGCUGCAGGCCUCCUUUUGCUGGCCGCUCAGCCCGCGAUGGCG** | **-3.2** |
| **Bottom-6** | **AAGUACUUAUUACCGACGGCAGCUGCGGGUCUGCUGCUCUUAGCUGCACAGCCAGCAAUGGCA** | **-3.2** |
| **Bottom-7** | **AAAUAUCUGCUACCUACUGCCGCAGCAGGUUUGCUAUUGCUUGCCGCACAGCCAGCCAUGGCG** | **-3.1** |
| **Bottom-8** | **AAGUACUUGUUGCCUACGGCGGCAGCAGGGCUAUUGCUCCUAGCUGCCCAGCCGGCCAUGGCG** | **-2.9** |
| **Bottom-9** | **AAGUACCUGCUCCCUACCGCAGCCGCAGGUUUACUUUUGCUGGCAGCACAGCCAGCGAUGGCA** | **-2.9** |
| **Bottom-10** | **AAAUACUUGCUCCCUACCGCAGCCGCUGGGCUUUUAUUGUUGGCGGCGCAGCCUGCGAUGGCU** | **-2.8** |

**Table S3** Plasmids used in this study

| **Plasmids** | **Description** | **Reference or source** |
| --- | --- | --- |
| pET-22b (+) | Containing signal peptide pelB for secretory expressing target protein, ampicillin^r^ | Merck KGaA, Darmstadt, Germany |
| pET-48b (+) | Containing Trx-Tag for promoting high-level expression, kanamycin^r^ | Merck KGaA, Darmstadt, Germany |
| pETDuet-1 | Control expression of two target genes under T7 promoter, ampicillin^r^ | Merck KGaA, Darmstadt, Germany |
| pCas | Cas9 expression vector, kanamycin^r^ | [1] |
| pTargetF | sgRNA expression vector, spectinomycin^r^ | [1] |
| pKJE7 | Encoding DnaK, DnaJ, and GrpE, chloromycetin^r^ | Takara, Dalian, China |
| pTf16 | Encoding Trigger factor, chloromycetin^r^ | Takara, Dalian, China |
| pET-22b/pro-TGm1 | The pET-22b derivative expressing TGm1 fused with the pro-region from *S. mobaraenesis* at N-terminus | This work |
| pETDuet/*TAMEP* | TAMEP expression plasmid | This work |
| pET-22b/*proC*-*TGm1* | The pET-22b derivative expressing TGm1 fused with the pro-region from *S. caniferus* at N-terminus | This work |
| pET-22b/*proF*-*TGm1* | The pET-22b derivative expressing TGm1 fused with the pro-region from *S. fradiae* at N-terminus | This work |
| pET-22b/*proH*-*TGm1* | The pET-22b derivative expressing TGm1 fused with the pro-region from *S. hygroscopicus* at N-terminus | This work |
| pET-22b/*proN*-*TGm1* | The pET-22b derivative expressing TGm1 fused with the pro-region from *S. netropsis* at N-terminus | This work |
| pET-22b/*proP*-*TGm1* | The pET-22b derivative expressing TGm1 fused with the pro-region from *S. platensis* at N-terminus | This work |
| pET-22b/*MBP*-*proH*-*TGm1* | The pET-22b derivative expressing TGm1 fused with fusion protein MBP and the pro-region from *S. platensis* at N-terminus | This work |
| pET-22b/*TrxA*-*proH*-*TGm1* | The pET-22b derivative expressing TGm1 fused with fusion protein TrxA and the pro-region from *S. platensis* at N-terminus | This work |
| pETDuet/*TrxA*-*proH*-*TGm1*/*TAMEP* | The pETDuet-1 derivative for co-expressing TrxA-proH-TGm1 and TAMEP | This work |
| pETDuet/*TrxA*-*proH*-*TGm1* | The pETDuet-1 derivative for expressing TrxA-proH-TGm1 | This work |
| pTargetF-*lpp* | Encoding sgRNA for *lpp* knockout | This work |

**Table S4** Synthetic double strand DNA fragment used in this study.

| Name | Sequences (5′-3′) | Remarks |
| --- | --- | --- |
| proC | AAGAAGGAGATATACATATGGCCAGCGGCGGCGACGAGGAATGGGAGGGGTCCTACGCCGCAACGCACGGCCTGACGGCGGAGGACGTCAAGAACATCAACGCACTGAACAAAAGGGCTCTGACTGCGGGTCAACCAGGCAATTTTCCGGCGGAATTGCCGCCGAGCGCCACTGCACTCTTCCGGGCCCCCGACGACCCAGACGACAGGGTC | Encoding pro-region of *S. caniferus* transglutaminase. |
| proF | AAGAAGGAGATATACATATGGCTGACTCAGGAGATGGTGCAAAAAAGGGGAGCTACGCGGAAACGCACGGCCTGACCGCACATGACGTTAAGAACATCAACGCCTTGAATGAGCGCGCTCCGGCGCTGGGCCAGCCGGGTAAACCGCCTGCGGGTGCGCCACCGTTCCGTACCCCGGCTGACCCAGACGACAGGGTC | Encoding pro-region of *S. fradiae* transglutaminase. |
| proH | AAGAAGGAGATATACATATGGCTAGCGGTGACGACGAGGAAAGGGAGGGGTCCTACGCCGAAACGCACGGTCTGACGGCGGAGGACGTCAAGAACATCAACGCACTGAACAAAAGGGCCCTGACTGCGGGTCAACCTGGCAATTCTCTGACGGAATTGCCGCCGAGCGTCAGTGCGCTCTTCCGGGCCCCCGACGACCCAGACGACAGGGTC | Encoding pro-region of *S. hygroscopicus* transglutaminase. |
| proN | AAGAAGGAGATATACATATGGCTGACGCGGGGGATGGTGCAAAAGAAGGAAGCTACGCGGAAACGCACGGCCTGACCGCACATGACGTTAAGAACATCAACGCCTTGAATGAGCGCGCGCTGGCTGTGGGCCAGCCGGGTAAACCGCCAGCGGGTGCTCCGCCGTTCCGTACCCCGGCGGACCCAGACGACAGGGTC | Encoding pro-region of *S. netropsis* transglutaminase. |
| proP | AAGAAGGAGATATACATATGGCCAGCCGCGGCGACGGGGAATGGGAAGGGTCCTACGCCGAAACGCACGGCCTGACGGCGGAGGATGTCAAGAACATCAACGCACTGAACAAAAGAGCTCTGACTGCGGGTCAACCCGGCAATTCTGCGGCGGAATTGCCGCCGAGCGCCAGTGCGCTCTTCCGGGCCCCCGACGACCCAGACGACAGGGTC | Encoding pro-region of *S. platensis* transglutaminase. |

The overlaps for homologous recombination were underlined.

**Table S5** Primers used in this study.

| Primers | Sequences (5′-3′) | Remarks |
| --- | --- | --- |
| ptgvf | GACCCAGACGACAGGGTCACCCCTC | Forward primer for generating vector for pro-region insertion using pET-22b/*pro*-*TGm1* as template. |
| ptgvr | CATATGTATATCTCCTTCTTAAAGTTAAAC | Reverse primer for generating vector for pro-region insertion using pET-22b/*pro*-*TGm1* as template. |
| ptrxf | AAGGAGATATACATATGAGCGATAAAATTATTCACCTGACTG | Forward primer for amplifying *TrxA* from pET-48b (+). |
| ptrxr | CGTCGTCACCGCTAGCGGCCAGGTTAGCGTCGAGGAACTCTTT | Reverse primer for amplifying *TrxA* from pET-48b (+). |
| ptmvf | GCTAGCGGTGACGACGAGGA | Forward primer for generating vector for *TrxA* and *MBP* insertion using pET-22b/*proH*-*TGm1* as template. |
| ptmvr | CATATGTATATCTCCTTCTTAAAGTTAAACAAAATTATTTCTAGAG | Reverse primer for generating vector for *TrxA* and *MBP* insertion using pET-22b/*proH*-*TGm1* as template. |
| pmbpf | AAGAAGGAGATATACATATGAAAATCGAAGAAGGTAAACTGGT | Forward primer for amplifying *MBP* from *E. coli* genome. |
| pmbpr | TCCTCGTCGTCACCGCTAGCAGTCTGCGCGTCTTTCAGGGCTTC | Reverse primer for amplifying *MBP* from *E. coli* genome. |
| pthtf | AGAAGGAGATATACCAGCGATAAAATTATTCACCTGACTGACGACA | Forward primer for amplifying *TrxA*-*proH*-*TGm1* from pET-22b/*TrxA*-*proH*-*TGm1*. |
| pthtr | GGCTTTAGTGGTGATGATGGTGATGTGGCCAGCCTT | Reverse primer for amplifying *TrxA*-*proH*-*TGm1* from pET-22b/*TrxA*-*proH*-*TGm1*. |
| pthtvf | TCACCACTAAAGCCAGGATCCGAATTCGAGCTC | Forward primer for generating vector for *TrxA*-*proH*-*TGm1* insertion using pETDuet/*TAMEP* as template. |
| pthtvr | GGTATATCTCCTTCTTAAAGTTAAAC | Reverse primer for generating vector for *TrxA*-*proH*-*TGm1* insertion using pETDuet/*TAMEP* as template. |
| ptampf | TAATTAACCTAGGCTGCTGCCACCGCTGAG | Forward primer for knockout *TAMEP* using pETDuet/*TrxA*-*proH*-*TGm1*/*TAMEP* as template. |
| ptampr | ATGTATATCTCCTTCTTATACTTAACTAATATACTAAGATGGG | Reverse primer for knockout *TAMEP* using pETDuet/*TrxA*-*proH*-*TGm1*/*TAMEP* as template. |
| pelbf | AGCGATAAAATTATTCACCTGACTGACGACA | Forward primer for generating vector for *pelB sequence variants* insertion using pETDuet/*TrxA*-*proH*-*TGm1*/*TAMEP* as template. |
| pelbr | GGTATATCTCCTTCTTAAAGTTAAAC | Reverse primer for generating vector for *pelB sequence variants* insertion using pETDuet/*TrxA*-*proH*-*TGm1*/*TAMEP* as template. |
| Top1f | GCTCTTAGCTGCGCAGCCAGCGATGGCGAGCGATAAAATTATTCACCTGACTGA | Forward primer for pelB sequence variants insertion using pETDuet/*TrxA*-*proH*-*TGm1*/*TAMEP* as template. |
| Top1r | AGGAGGCCGGCCGCTGCTGTGGGCAATAAATATTTGGTATATCTCCTTCTTAAAGTTAAAC | Reverse primer for pelB sequence variants insertion using pETDuet/*TrxA*-*proH*-*TGm1*/*TAMEP* as template. |
| Top2f | TTCTTTTGTTAGCCGCGCAGCCGGCTATGGCAAGCGATAAAATTATTCACCTGACTGA | Forward primer for pelB sequence variants insertion using pETDuet/*TrxA*-*proH*-*TGm1*/*TAMEP* as template. |
| Top2r | GGCCAGCAGCAGCGGTGGGAAGGAGATATTTGGTATATCTCCTTCTTAAAGTTAAAC | Reverse primer for pelB sequence variants insertion using pETDuet/*TrxA*-*proH*-*TGm1*/*TAMEP* as template. |
| Top3f | TACTTCTTGCAGCCCAACCCGCCATGGCTAGCGATAAAATTATTCACCTGACTGA | Forward primer for pelB sequence variants insertion using pETDuet/*TrxA*-*proH*-*TGm1*/*TAMEP* as template. |
| Top3r | GCAGCCCTGCAGCGGCCGTTGGCAAAAGGTATTTGGTATATCTCCTTCTTAAAGTTAAAC | Reverse primer for pelB sequence variants insertion using pETDuet/*TrxA*-*proH*-*TGm1*/*TAMEP* as template. |
| Top4f | TTTTGCTACTCGCTGCGCAGCCCGCCATGGCTAGCGATAAAATTATTCACCTGACTGA | Forward primer for pelB sequence variants insertion using pETDuet/*TrxA*-*proH*-*TGm1*/*TAMEP* as template. |
| Top4r | GTCCGGCCGCTGCAGTAGGAAGCAAATACTTGGTATATCTCCTTCTTAAAGTTAAAC | Reverse primer for pelB sequence variants insertion using pETDuet/*TrxA*-*proH*-*TGm1*/*TAMEP* as template. |
| Top5f | CTACTACTCTTGGCCGCACAACCCGCCATGGCAAGCGATAAAATTATTCACCTGACTGA | Forward primer for pelB sequence variants insertion using pETDuet/*TrxA*-*proH*-*TGm1*/*TAMEP* as template. |
| Top5r | TCCCGCAGCAGCAGTCGGGAGGAGGTACTTGGTATATCTCCTTCTTAAAGTTAAAC | Reverse primer for pelB sequence variants insertion using pETDuet/*TrxA*-*proH*-*TGm1*/*TAMEP* as template. |
| Top6f | TGGCCTGCTTCTCCTCGCCGCCCAGCCCGCAATGGCCAGCGATAAAATTATTCACCTGACTGA | Forward primer for pelB sequence variants insertion using pETDuet/*TrxA*-*proH*-*TGm1*/*TAMEP* as template. |
| Top6r | GCTGCTGCGGTAGGGAGTAGGTATTTGGTATATCTCCTTCTTAAAGTTAAAC | Reverse primer for pelB sequence variants insertion using pETDuet/*TrxA*-*proH*-*TGm1*/*TAMEP* as template. |
| Top7f | CTCTTGTTACTCGCCGCCCAGCCAGCAATGGCGAGCGATAAAATTATTCACCTGACTGA | Forward primer for pelB sequence variants insertion using pETDuet/*TrxA*-*proH*-*TGm1*/*TAMEP* as template. |
| Top7r | ACCAGCCGCTGCGGTGGGAAGGAGATATTTGGTATATCTCCTTCTTAAAGTTAAAC | Reverse primer for pelB sequence variants insertion using pETDuet/*TrxA*-*proH*-*TGm1*/*TAMEP* as template. |
| Top8f | TGCTACTCGCGGCACAGCCTGCTATGGCTAGCGATAAAATTATTCACCTGACTGA | Forward primer for pelB sequence variants insertion using pETDuet/*TrxA*-*proH*-*TGm1*/*TAMEP* as template. |
| Top8r | AAAGCCCTGCGGCCGCCGTCGGTAAAAGATACTTGGTATATCTCCTTCTTAAAGTTAAAC | Reverse primer for pelB sequence variants insertion using pETDuet/*TrxA*-*proH*-*TGm1*/*TAMEP* as template. |
| Top9f | CCGGCCGCAGCTGTAGGTAGTAGATACTTGGTATATCTCCTTCTTAAAGTTAAAC | Forward primer for pelB sequence variants insertion using pETDuet/*TrxA*-*proH*-*TGm1*/*TAMEP* as template. |
| Top9r | CCTGCTCTTACTAGCAGCTCAGCCGGCGATGGCAAGCGATAAAATTATTCACCTGACTGA | Reverse primer for pelB sequence variants insertion using pETDuet/*TrxA*-*proH*-*TGm1*/*TAMEP* as template. |
| Top10f | CCTGCTCTTGTTGGCTGCACAACCCGCTATGGCAAGCGATAAAATTATTCACCTGACTGA | Forward primer for pelB sequence variants insertion using pETDuet/*TrxA*-*proH*-*TGm1*/*TAMEP* as template. |
| Top10r | CCGGCCGCCGCCGTCGGTAGTAAGTATTTGGTATATCTCCTTCTTAAAGTTAAAC | Reverse primer for pelB sequence variants insertion using pETDuet/*TrxA*-*proH*-*TGm1*/*TAMEP* as template. |
| Bot1f | TTTTATTGTTGGCGGCGCAGCCTGCGATGGCTAGCGATAAAATTATTCACCTGACTGA | Forward primer for pelB sequence variants insertion using pETDuet/*TrxA*-*proH*-*TGm1*/*TAMEP* as template. |
| Bot1r | GCCCAGCGGCTGCGGTAGGGAGCAAGTATTTGGTATATCTCCTTCTTAAAGTTAAAC | Reverse primer for pelB sequence variants insertion using pETDuet/*TrxA*-*proH*-*TGm1*/*TAMEP* as template. |
| Bot2f | TTTGCTGGCAGCACAGCCAGCGATGGCAAGCGATAAAATTATTCACCTGACTGA | Forward primer for pelB sequence variants insertion using pETDuet/*TrxA*-*proH*-*TGm1*/*TAMEP* as template. |
| Bot2r | AGTAAACCTGCGGCTGCGGTAGGGAGCAGGTACTTGGTATATCTCCTTCTTAAAGTTAAAC | Reverse primer for pelB sequence variants insertion using pETDuet/*TrxA*-*proH*-*TGm1*/*TAMEP* as template. |
| Bot3f | CTATTGCTCCTAGCTGCCCAGCCGGCCATGGCGAGCGATAAAATTATTCACCTGACTGA | Forward primer for pelB sequence variants insertion using pETDuet/*TrxA*-*proH*-*TGm1*/*TAMEP* as template. |
| Bot3r | CCCTGCTGCCGCCGTAGGCAACAAGTACTTGGTATATCTCCTTCTTAAAGTTAAAC | Reverse primer for pelB sequence variants insertion using pETDuet/*TrxA*-*proH*-*TGm1*/*TAMEP* as template. |
| Bot4f | TTTGCTATTGCTTGCCGCACAGCCAGCCATGGCGAGCGATAAAATTATTCACCTGACTGA | Forward primer for pelB sequence variants insertion using pETDuet/*TrxA*-*proH*-*TGm1*/*TAMEP* as template. |
| Bot4r | CCTGCTGCGGCAGTAGGTAGCAGATATTTGGTATATCTCCTTCTTAAAGTTAAAC | Reverse primer for pelB sequence variants insertion using pETDuet/*TrxA*-*proH*-*TGm1*/*TAMEP* as template. |
| Bot5f | TGCTGCTCTTAGCTGCACAGCCAGCAATGGCAAGCGATAAAATTATTCACCTGACTGA | Forward primer for pelB sequence variants insertion using pETDuet/*TrxA*-*proH*-*TGm1*/*TAMEP* as template. |
| Bot5r | GACCCGCAGCTGCCGTCGGTAATAAGTACTTGGTATATCTCCTTCTTAAAGTTAAAC | Reverse primer for pelB sequence variants insertion using pETDuet/*TrxA*-*proH*-*TGm1*/*TAMEP* as template. |
| Bot6f | TGCAGGCCTCCTTTTGCTGGCCGCTCAGCCCGCGATGGCGAGCGATAAAATTATTCACCTGACTGA | Forward primer for pelB sequence variants insertion using pETDuet/*TrxA*-*proH*-*TGm1*/*TAMEP* as template. |
| Bot6r | GCTGCGGTGGGGAGGAGATATTTGGTATATCTCCTTCTTAAAGTTAAAC | Reverse primer for pelB sequence variants insertion using pETDuet/*TrxA*-*proH*-*TGm1*/*TAMEP* as template. |
| Bot7f | GCTGCTTCTAGCTGCACAGCCGGCCATGGCTAGCGATAAAATTATTCACCTGACTGA | Forward primer for pelB sequence variants insertion using pETDuet/*TrxA*-*proH*-*TGm1*/*TAMEP* as template. |
| Bot7r | AAGCCGGCAGCGGCGGTAGGTAGCAGGTATTTGGTATATCTCCTTCTTAAAGTTAAAC | Reverse primer for pelB sequence variants insertion using pETDuet/*TrxA*-*proH*-*TGm1*/*TAMEP* as template. |
| Bot8f | GTTTGCTTCTCCTGGCTGCGCAGCCTGCGATGGCCAGCGATAAAATTATTCACCTGACTGA | Forward primer for pelB sequence variants insertion using pETDuet/*TrxA*-*proH*-*TGm1*/*TAMEP* as template. |
| Bot8r | CCGCAGCAGCGGTGGGAAGCAGGTACTTGGTATATCTCCTTCTTAAAGTTAAAC | Reverse primer for pelB sequence variants insertion using pETDuet/*TrxA*-*proH*-*TGm1*/*TAMEP* as template. |
| Bot9f | GGGTTATTACTACTTGCTGCGCAGCCAGCAATGGCAAGCGATAAAATTATTCACCTGACTGA | Forward primer for pelB sequence variants insertion using pETDuet/*TrxA*-*proH*-*TGm1*/*TAMEP* as template. |
| Bot9r | GGCAGCAGCTGTAGGCAGTAGGTACTTGGTATATCTCCTTCTTAAAGTTAAAC | Reverse primer for pelB sequence variants insertion using pETDuet/*TrxA*-*proH*-*TGm1*/*TAMEP* as template. |
| Bot10f | TGCTACTTTTGGCCGCCCAGCCCGCCATGGCAAGCGATAAAATTATTCACCTGACTGA | Forward primer for pelB sequence variants insertion using pETDuet/*TrxA*-*proH*-*TGm1*/*TAMEP* as template. |
| Bot10r | ACCCGGCGGCGGCGGTGGGCAACAGATACTTGGTATATCTCCTTCTTAAAGTTAAAC | Reverse primer for pelB sequence variants insertion using pETDuet/*TrxA*-*proH*-*TGm1*/*TAMEP* as template. |
| lppupf | GACAACCGTAAACTGCGCATTACCGAAG | Forward primer for amplifying upstream DNA flanked *lpp* site. |
| lppupr | CAAGGGAATATGTTACAACAGGATGCTTCCATCGGATTCATCTT | Reverse primer for amplifying upstream DNA flanked *lpp* site. |
| lppdownf | AAGCATCCTGTTGTAACATATTCCCTTGCTCTGGTTCACCA | Forward primer for amplifying downstream DNA flanked *lpp* site. |
| lppdownr | TGGCATTAAATTACCGCCAGTCGTTCCT | Reverse primer for amplifying downstream DNA flanked *lpp* site. |
| ptrf | TAACCAGCGTCTGGACAACAGTTTTAGAGCTAGAAATAGCAAGTT | Forward primer for architecting pTargetF-*lpp*. |
| ptrr | TGTTGTCCAGACGCTGGTTAACTAGTATTATACCTAGGACTGAG | Reverse primer for architecting pTargetF-*lpp*. |
| VF | ATCCGCAAAAGGTAAATACCCGCTGGAAG | Forward primer for colony PCR after pCas and pTargetF-*lpp* transformed into *E. coli*. |
| VR | AAACGCGTGTTGGGCAGAAAATCCCT | Reverse primer for colony PCR after pCas and pTargetF-*lpp* transformed into *E. coli*. |

The overlaps for homologous recombination were underlined.

**Figures**

**Figure S1** Expression of TGm1 in different forms. *E. coli* transformed with (A) pET-22b/pro-TGm1, (B) pET-22b/proH-TGm1, and (C) pET-22b-trxA-proH-TGm1. 1, 3, and 5: insoluble fractions; 2, 4, and 6: soluble fractions. *E. coli* was cultivated at 20 ℃ for 32 h after induction by adding IPTG at a final concentration of 0.1 mM when OD_600_ reached 1.0. Red arrow: (A) pro-TGm1, (B) proH-TGm1, and (C) TrxA-proH-TGm1.

**Figure S2** SDS-PAGE analysis of smTG variants purified by Nickel affinity chromatography. 1: FRAP-TGm1; 2: FRAPD-TGm1;

**Figure S3** Edman sequencing the N-terminus of recombinant TGm1 variants activated by dispase *in vitro*. pro-TGm1 and proH-TGm1 were intracellularly expressed in *E*. *coli* using pET-22b/*pro-TGm1* and pET-22b/*proH-TGm1*, recombinantly expressed TGm1 variants were *in vitro* activated and purified by affinity chromatography using HisTrap (GE Healthcare, USA) column.

**Figure S4** *E. coli* carrying pETDuet/*pelB*-*TrxA*-*proH*-*TGm1* /*TAMEP* and pKJE7 was cultivated under 20 ℃ after induction by IPTG.

**Figure S5** MD simulation of FRAP-TGm1 and FRAPD-TGm1. **A** Hydrogen bonds within the N-terminus (FRAPDPDD) of FRAP-TGm1. **B** Hydrogen bonds within the N-terminus (FRAPDPDD) of FRAPD-TGm1. MD simulation was conducted for 100 ns using Gromacs-2020. **C** The modeled structure of FRAPD-TGm1. The N-terminal loop of FRAPD-TGm1 was colored in cyan.

**Figure S6** Sequence analysis of pro-TGm1 (GenBank ID: MZ516369). The number of nucleotide sequences (left side); Single underline: *pro-region*; Double underline: mature region of *TGm1*; Box, His-tag.

**Figure S7** Sequence analysis of TAMEP (GenBank ID: MZ516816). A start codon (ATG) was placed after the predicted signal peptide. Single underline: signal peptide [2]. Numbers (left side): the number of nucleotide sequences. Box, His-tag. For purification purpose, a His-tag was placed as shown in box while constructing the plasmid pETDuet/TAMEP; for co-expressing TAMEP with TGm1, the His-tag (as shown in box) was removed.

**References:**

1. Jiang Y, Chen B, Duan C, Sun B, Yang J, Yang S, Kelly RM: Multigene editing in the *Escherichia coli* genome via the CRISPR-Cas9 system. Appl Environ Microbiol. 2015, 81:2506-2514.

2. Juettner NE, Classen M, Colin F, Hoffmann SB, Meyners C, Pfeifer F, Fuchsbauer H-L: Features of the transglutaminase-activating metalloprotease from *Streptomyces mobaraensis* DSM 40847 produced in *Escherichia coli*. J Biotechnol. 2018, 281:115-122.
